# Functional Conservation of Coenzyme Q Biosynthetic Genes among Yeasts, Plants, and Humans

**DOI:** 10.1371/journal.pone.0099038

**Published:** 2014-06-09

**Authors:** Kazuhiro Hayashi, Yuki Ogiyama, Kazumasa Yokomi, Tsuyoshi Nakagawa, Tomohiro Kaino, Makoto Kawamukai

**Affiliations:** 1 Department of Life Science and Biotechnology, Faculty of Life and Environmental Science, Shimane University, Matsue, Japan; 2 Interdisciplinary Center for Science Research, Shimane University, Matsue, Japan; Cancer Research UK London Research Institute, United Kingdom

## Abstract

Coenzyme Q (CoQ) is an essential factor for aerobic growth and oxidative phosphorylation in the electron transport system. The biosynthetic pathway for CoQ has been proposed mainly from biochemical and genetic analyses of *Escherichia coli* and *Saccharomyces cerevisiae*; however, the biosynthetic pathway in higher eukaryotes has been explored in only a limited number of studies. We previously reported the roles of several genes involved in CoQ synthesis in the fission yeast *Schizosaccharomyces pombe*. Here, we expand these findings by identifying ten genes (*dps1, dlp1, ppt1*, and *coq3–9*) that are required for CoQ synthesis. CoQ10-deficient *S. pombe coq* deletion strains were generated and characterized. All mutant fission yeast strains were sensitive to oxidative stress, produced a large amount of sulfide, required an antioxidant to grow on minimal medium, and did not survive at the stationary phase. To compare the biosynthetic pathway of CoQ in fission yeast with that in higher eukaryotes, the ability of CoQ biosynthetic genes from humans and plants (*Arabidopsis thaliana*) to functionally complement the *S. pombe coq* deletion strains was determined. With the exception of *COQ9*, expression of all other human and plant *COQ* genes recovered CoQ10 production by the fission yeast *coq* deletion strains, although the addition of a mitochondrial targeting sequence was required for human *COQ3* and *COQ7*, as well as *A. thaliana COQ6*. In summary, this study describes the functional conservation of CoQ biosynthetic genes between yeasts, humans, and plants.

## Introduction

Coenzyme Q (CoQ), also known as ubiquinone, is an isoprenoid quinone that is distributed widely in almost all living organisms [1]–[3]. CoQ is a component of the respiratory chain in the inner mitochondrial membrane of eukaryotes and functions primarily as an electron transporter during aerobic respiration and oxidative phosphorylation. CoQ serves as the electron transporter of the NADH dehydrogenase and succinate dehydrogenase complexes to form CoQ:cytochrome *c* reductase and thus is an essential component of the ATP synthesis pathway. CoQ also functions as a lipid-soluble antioxidant that scavenges reactive oxygen species in cellular biomembranes [4]. Additional roles of CoQ include disulfide bond formation [5], sulfide oxidation [6], and pyrimidine metabolism [7], [8].

**Figure 1:**
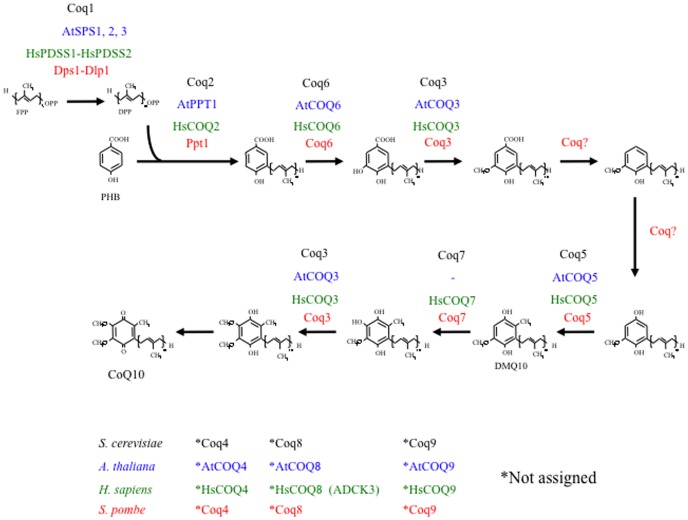
The proposed CoQ biosynthesis pathway in *S. cerevisiae*, *S. pombe*, humans, and *A. thaliana*. At least ten genes, three of which have unassigned roles, are responsible for CoQ biosynthesis in *S. pombe*. All of these *S. pombe* enzymes have human counterparts, but *A. thaliana* lacks Coq7 and a component of the prenyl diphosphate synthase in this plant species differs from that in *S. pombe* and humans. The functions of Coq4 and Coq9 are currently unknown. Coq8 is a protein kinase that regulates the stability of Coq proteins in *S. cerevisiae*. The involvement of pABA in the CoQ pathway in *S. pombe*, human and *A. thaliana* has not yet been established. For simplicity, *ARH1* and *YAH1*, which are involved in CoQ synthesis through the regulation of Coq6 in *S. cerevisiae*, are not shown.

In living organisms, CoQ exists in a number of different forms with differing isoprenoid side chain lengths. For example, in humans and the fission yeast *Schizosaccharomyces pombe*, the CoQ side chain comprises ten isoprene units (CoQ10), whereas those in *Arabidopsis thaliana* and *Saccharomyces cerevisiae* are composed of nine (CoQ9) and six (CoQ6) units, respectively [1]. The length of the side chain is defined by *trans*-polyprenyl diphosphate synthases rather than by the *p*-hydroxybenzoate (PHB)-polyprenyl diphosphate transferases that catalyze the condensation of PHB and polyprenyl diphosphate [9]. Synthesis of CoQ occurs in two stages: the synthesis of isoprenoid and the synthesis of quinone (Figure 1). To synthesize the isoprenoid tail, a unit of prenyl diphosphate is synthesized by polyprenyl diphosphate synthase. In *S. pombe* and humans, this enzyme is a heterotetramer of decaprenyl diphosphate synthase (Dps1; also known as Pdss1) and D (aspartate)-less polyprenyl diphosphate synthase (Dlp1; also known as Pdss2) [10]–[12]. By contrast, in budding yeast and plants, polyprenyl diphosphate synthase is homomeric (presumably a homodimer) [13]–[16]. Although the synthesis of isoprenoid has been characterized, the mechanisms involved in quinone synthesis in eukaryotes are less well known. In *S. cerevisiae*, the biosynthetic pathway that converts PHB to CoQ comprises at least eight steps that require at least seven enzymes with assigned roles [1], [2], [17]; these steps include the condensation and transfer of the isoprenoid side chain to PHB, followed by methylations, decarboxylation and hydroxylations (Figure 1). Para-aminobenzoic acid (pABA) is also a precursor of CoQ biosynthesis in budding yeast [18]. PHB-polyprenyl diphosphate transferase, known as Coq2 in budding yeast [19] or Ppt1 in fission yeast [20], catalyzes the condensation of PHB (or pABA) with the isoprenoid chain. Coq3 (*O*-methyltransferase) catalyzes the two *O*-methylation steps in the CoQ biosynthetic pathway [21], [22]. Coq4 is absolutely required for CoQ biosynthesis but its enzymatic function remains unknown [23]. Coq5 (*C*-methyltransferase) catalyzes the only *C*-methylation step in the pathway [24]. Coq6 is a flavin-dependent monooxygenase responsible for adding the hydroxy group to polyprenyl-PHB at the C5 position [25], [26]. The mono-oxygenase Coq7 is involved in the penultimate step of CoQ biosynthesis [22]. Coq8 functions as a protein kinase that phosphorylates some Coq proteins and stabilizes the protein complex [27], [28]. Coq9 is required for CoQ biosynthesis but its enzymatic function is unknown [29]. Coq10 is a binding protein of CoQ [30], [31], and indirectly affects but is not required for CoQ synthesis [32].

Although the CoQ biosynthetic pathway has been examined in bacteria and yeasts, little is known about the pathway in higher eukaryotes. Understanding the CoQ biosynthetic pathway in humans is important because CoQ is essential for energy production and is the only endogenously synthesized lipid-soluble antioxidant. Furthermore, CoQ deficiency can lead to the development of severe diseases such as Leigh syndrome [33].

Here, we identified ten genes (*dps1, dlp1, ppt1*, and *coq3*–*9*), including some that have been reported previously, that are related to CoQ biosynthesis in *S. pombe*. Deletion strains were constructed for all of these genes; all of the CoQ10-deficient mutants were sensitive to oxidative stress, produced a large quantity of sulfide, required an antioxidant to grow on minimal medium, and did not survive in the stationary phase. The biosynthetic pathway of CoQ in higher eukaryotes was also investigated by performing functional complementation analyses of the *S. pombe coq* deletion mutants using human and *A. thaliana* homologues.

## Materials and Methods

### Strains, media, and genetic manipulation

The *S. pombe* strains used in this study are listed in Table 1. Standard yeast culture media and genetic manipulations were used. The *S. pombe* strains were grown in complete YES medium (0.5% yeast extract, 3% glucose, 225 mg/l adenine, 225 mg/l leucine, 225 mg/l uracil, 225 mg/l histidine, and 225 mg/l lysine hydrochloride) or EMM medium (0.3% potassium hydrogen phthalate, 0.56% sodium phosphate, 0.5% ammonium chloride, 2% glucose, vitamins, minerals, and salts) [34]. The appropriate auxotrophic supplements were added as necessary (75 mg/l adenine, 75 mg/l leucine, 75 mg/l uracil, 75 mg/l histidine, 75 mg/l lysine, or 400 mg/l cysteine). *Escherichia coli* DH5α was used as the host strain for all plasmid manipulations and was grown in LB medium (1% bactotryptone, 0.5% yeast extract, and 1% NaCl; pH 7.0). Standard molecular biology techniques were followed [35]. The restriction enzymes (*Bam*HI, *Bgl*II, *Xho*I, *Nde*I, *Not*I and *Sal*I) were used according to the suppliers' recommendations (TOYOBO, Takara, NEB). Nucleotide sequences were determined by the dideoxynucleotide chain-termination method using an Applied Biosystems 3500 Genetic Analyzer.

**Table 1:** Strains used in this study.

Strain	Genotype	Resource
L972	*h˜*	Lab stock
PR110	*h* ^+^ *leu1-32 ura4-D18*	Lab stock
LJ1030	*h* ^+^ *leu1-32 ura4-D18 dps1:: kanMX*	[6]
RM19	*h* ^+^ *leu1-32 ura4-D18 dlp1:: kanMX6*	[22]
LA1	*h* ^+^ *leu1-32 ura4-D18 ade6.M210 dps1:: kanMX6 dlp1::ura4::ADE2*	[6]
KH2	*h* ^+^ *leu1-32 ura4-D18 ppt1(coq2)::kanMX6*	This study
KH3 (RM2)	*h* ^+^ *leu1-32 ura4-D18 coq3:: kanMX6*	[22]
KH4	*h* ^+^ *leu1-32 ura4-D18 coq4:: kanMX6*	This study
KH5	*h* ^+^ *leu1-32 ura4-D18 coq5:: kanMX6*	This study
KH6	*h* ^+^ *leu1-32 ura4-D18 coq6:: kanMX6*	This study
KH7 (RM1)	*h* ^+^ *leu1-32 ura4-D18 coq7:: kanMX6*	[22]
KH8	*h* ^+^ *leu1-32 ura4-D18 coq8:: kanMX6*	This study
KH9	*h* ^+^ *leu1-32 ura4-D18 coq9:: kanMX6*	This study

### Construction of the deletion strains

The *S. pombe* deletion strains were constructed by replacing the *coq* genes with a selectable marker; chromosomal genes were disrupted by homologous recombination using fragments generated by polymerase chain reaction (PCR) [36] (Figure S1). The 1.6 kb *kanMX6* module was amplified with flanking sequences corresponding to the 5′ and 3′ ends of the target genes. Resistant colonies were selected on YES plates containing 100 mg/L G418 (Sigma) and disruption of the gene of interest was verified by colony PCR. DNA fragments of 400–500 bp corresponding to the 5' or 3' regions of the *ppt1*, *coq4, coq5, coq6, coq8*, or *coq9* gene were amplified by PCR using the ppt1/coq4/5/6/8/9-w and ppt1/coq4/5/6/8/9-x or ppt1/coq4/5/6/8/9-y and ppt1/coq4/5/6/8/9-z primer pairs (Table S1). For each gene, the 5′ and 3′ amplified fragments were fused to the ends of the *kanMX6* module by PCR. The wild type PR110 strain (Table 1) was transformed with the resulting *ppt1::kanMX6*, *coq4::kanMX6, coq5::kanMX6, coq6::kanMX6, coq8::kanMX6*, and *coq9::kanMX6* fragments to form the deletion strains (Table 1). Transformants were selected using 100 mg/L G418. To confirm the chromosomal deletion of the *ppt1, coq4, coq5, coq6, coq8*, and *coq9* genes, PCR was performed using the nb2 and ppt1/coq4/5/6/8/9-c primers (Table S1); the resulting deletion strains were designated KH2 (Δ*ppt1*), KH4 (Δ*coq4*), KH5 (Δ*coq5*), KH6 (Δ*coq6*), KH8 (Δ*coq8*), and KH9 (Δ*coq9*), respectively. The Δ*dps1* strain and the *dps1-dlp1* double deletion strain were constructed as described previously (Zhang et al., 2008). The Δ*dlp1*, Δ*coq3* and Δ*coq7* strains were also constructed as described previously (Miki et al., 2008).

### Plasmid construction

The plasmids constructed in this study are shown in Figures S2, S3, Figure S4 and S5 and the primers used for plasmid construction are listed in Table S1. Each *S. pombe coq* gene was PCR amplified using primers containing restriction sites, digested using restriction endonucleases, and then inserted into the appropriate sites of the pREP1 or pREP2 vector by ligation. For example, pREP1-coq3 was constructed by inserting the PCR product amplified using the Coq3-N (SalI) and Coq3-C (SmaI) primers into the *Sal*I and *Sma*I sites of pREP1 (Figure S2). The other plasmids were constructed similarly. To examine the cellular localization of Coq proteins, GFP-fusions were generated by inserting each *coq* gene into the pSLF272L-GFP_S65A_ vector. For example, pSLF272L-GFP_S65A_-Coq3 was constructed by inserting the PCR product amplified using the Coq3-N-GFP (XhoI) and Coq3-C-GFP (NotI) primers into the *Xho*I and *Not*I sites of pSLF272L-GFP_S65A_. The other plasmids expressing GFP-fusion proteins were constructed similarly (Figure S3). The human (Hs) *COQ* genes (Table 2) were obtained from Funakoshi Corp. or from C. Clarke at UCLA. The DNA fragments were amplified by PCR using primers containing restriction sites (Table S1) and then cloned into the pREP1, pREP41, or pREP2 vectors [37]. For example, pREP1-HsCOQ3 was constructed by inserting the PCR product amplified using the HsCOQ3-N (NdeI) and HsCOQ3-C (BamHI) primers into the *Nde*I and *Bam*HI sites of pREP1 (Figure S4). For expression of Hs*COQ3* and Hs*COQ7*, the 5′ region and mitochondrial targeting sequence of *coq3* and *coq7* from *S. pombe* were fused with the human *COQ3* and *COQ7* sequences, respectively. The *A. thaliana* genes (Table 2) were obtained from the Arabidopsis Biological Resource Center (ABRC) or Riken BRC. The plasmids were constructed in the same manner as those expressing the human *COQ3* genes. For expression of *AtCOQ6*, the mitochondrial targeting signal of *coq6* from *S. pombe* was fused to the *AtCOQ6* gene sequence (Figure S5). The genes amplified by PCR were verified by DNA sequencing.

**Table 2:** Genes involved in CoQ synthesis in *S. cerevisiae, S. pombe*, humans, and *A. thaliana*.

*S. cerevisiae*	*S. pombe*	*H. sapiens* (accession #)	*Arabidopsis* (systematic name)	Enzyme (function)
*COQ1*	*dps1-dlp1*	Hs*PDSS1*(AI590245)-*HsPDSS2*(BI551760)	At*SPS3* (*At2g34630*)	Polyprenyl diphosphate synthase
*COQ2*	*ppt1/coq2*	Hs*COQ2* (BF026793)	At*PPT1* (*At4g23660*)	PHB-polyprenyldiphosphate transferase
*COQ3*	*coq3*	Hs*COQ3* (AF193016)	At*COQ3* (*At2g30920*)	O-Methyltransferase
*COQ4*	*coq4*	Hs*COQ4* (BQ685658)	At*COQ4* (*At2g03690*)	Unknown
*COQ5*	*coq5*	Hs*COQ5* (BC004916)	At*COQ5* (*At5g57300*)	C-Methyltransferase
*COQ6*	*coq6*	Hs*COQ6* (BQ668512)	At*COQ6* (*At3g24200*)	C5-Hydroxylation
*COQ7*	*coq7*	Hs*COQ7* (AF032900)	-	Monooxygenase
*COQ8/ABC1*	*coq8*	Hs*COQ8*/ADCK3 (BC005171)	At*COQ8* (*At4g01660*)	Protein kinase
*COQ9*	*coq9*	Hs*COQ9* (BC064946)	At*COQ9* (*At1g19140*)	Unknown

### Extraction and measurement of CoQ from *S. pombe*

CoQ was extracted from *S. pombe* as described previously [31]. Briefly, crude lipid extracts were analyzed by normal phase thin layer chromatography using authentic CoQ10 (as the standard) and benzene on a Kieselgel 60 F_254_ plate. Following UV visualization, the band containing CoQ10 was collected from the plate and extracted with chloroform/methanol (1∶1 v/v). The samples were dried and resolved in ethanol. Purified CoQ was analyzed further by high-performance liquid chromatography (HPLC), with ethanol as the solvent.

### Measurement of extracellular sulfide

Extracellular sulfide was quantified using the methylene blue method [6]. Briefly, *S. pombe* was grown to late log phase in YES or EMM medium (50 ml). The cultures (0.5 ml) were mixed with 0.1 ml of 0.1% dimethylphenylenediamine (in 5.5 N HCl) and 0.1 ml of 23 mM FeCl_3_ (in 1.2 N HCl) and then incubated at 37°C for 5 min. Following centrifugation at 12,000 g for 5 min, the supernatant was removed and absorbance was measured at 670 nm. The blank consisted of reagents alone.

### Mitochondrial staining and fluorescence microscopy

Mitochondria were stained using MitoTracker Red FM dye (Invitrogen). The cells were suspended in 10 mM HEPES (pH 7.4) containing 5% glucose and MitoTracker Red FM was added to a final concentration of 50 nM. After incubation at room temperature for 15 min, the cells were visualized at 1000× magnification using a BX51 fluorescent microscope (Olympus, Tokyo, Japan). The fluorescence of GFP_S65A_ was observed at an excitation wavelength of 485 nm. Fluorescent images were obtained using a digital camera (DP70, Olympus) connected to the microscope.

## Results

### Construction and phenotypes of the *S. pombe coq* deletion strains

The biosynthetic pathway of CoQ has been well characterized in *S. cerevisiae* [2]. Here, we examined the involvement of ten *S. pombe coq* genes (which are homologous to *S. cerevisiae COQ* genes) (Figure 1 and Table 2) in CoQ biosynthesis. We previously reported the construction and characterization of *S. pombe Δdps1* [6], [38], *Δdlp1* [11], *Δdps1-Δdlp1* (Zhang et al., 2008), *Δppt1*/*coq2* [20], *Δcoq3* [22], *Δcoq7* [22], and *Δcoq8* [27] strains, which are unable to synthesize CoQ. Here, we constructed four new *S. pombe* deletion strains in which the *coq4* (*SPAC1687.12c*), *coq5* (*SPCC4G3.04c*), *coq6* (*SPBC146.12*), or *coq9* (*SPAC19G12.11*) gene was replaced with the *kan* ^r^ marker (Figure S1 and Table 1). *S. pombe ppt1* or *coq8* deletion strain containing the *kan* ^r^ marker was also generated to equalize a background of strains.

**Figure 2:**
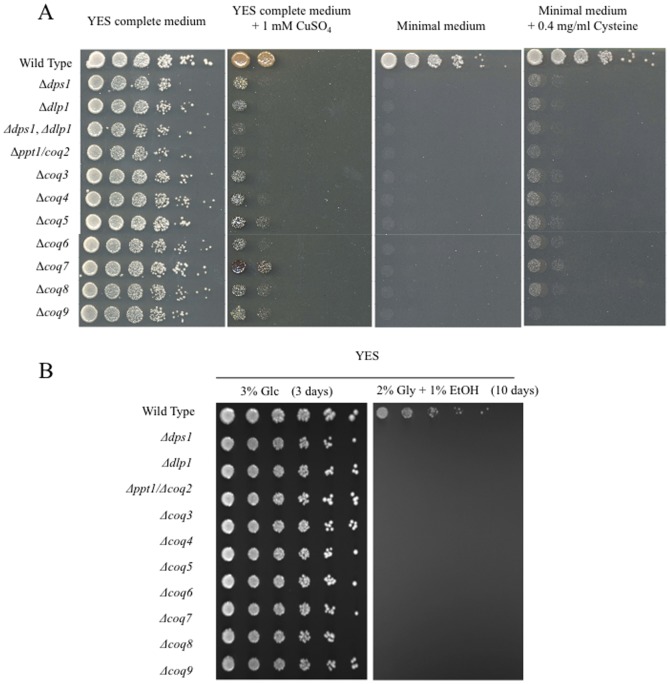
Phenotypes of the *S. pombe coq* deletion mutants. (A) Ten-fold serial dilutions of the indicated strains (1×10^6^ cells) in sterilized water were spotted onto YES medium, YES medium supplemented with 1 mM CuSO_4_, EMM (minimal medium), and EMM supplemented with 0.4 mg/ml cysteine. The plates were incubated at 30°C for 4 days (B) The same dilutions of the strains were spotted onto YES medium containing 3 mM glucose (Glc) and YES medium lacking glucose but containing 2% glycerol (Gly) and 1% ethanol (EtOH). The plates were incubated at 30°C for 3 or 10 days.

The phenotypes of eleven *S. pombe* deletion strains (*Δdps1*, *Δdlp1*, *Δdps1-Δdlp1*, *Δppt1*/*coq2*, *Δcoq3*, *Δcoq4*, *Δcoq5*, *Δcoq6*, *Δcoq7*, *Δcoq8*, and *Δcoq9*; Table 1) were determined. Unlike the wild type strain (PR110), all of the deletion strains required cysteine to grow on minimal medium and were sensitive to CuSO_4_ when grown on YES medium (Figure 2A). In addition, the ten single deletion strains (the *Δdps1-Δdlp1* strain was not tested) were unable to grow on YES medium containing yeast extract supplemented with ethanol and glycerol (Figure 2B) and did not survive in the stationary phase (data not shown; [22]). The inability to grow on YES medium containing yeast extract supplemented with ethanol and glycerol and the sensitivity to CuSO_4_ are symptoms of respiration defective and oxidative stress-sensitive phenotypes, respectively. As reported previously for the *S. pombe Δcoq7* strain [22], all ten single *coq* deletion strains were also sensitive to H_2_O_2_ (data not shown). Overall, these results suggest that the ten single *coq* deletion strains share common phenotypes.

### Localization of the *S. pombe* Coq proteins

**Figure 3:**
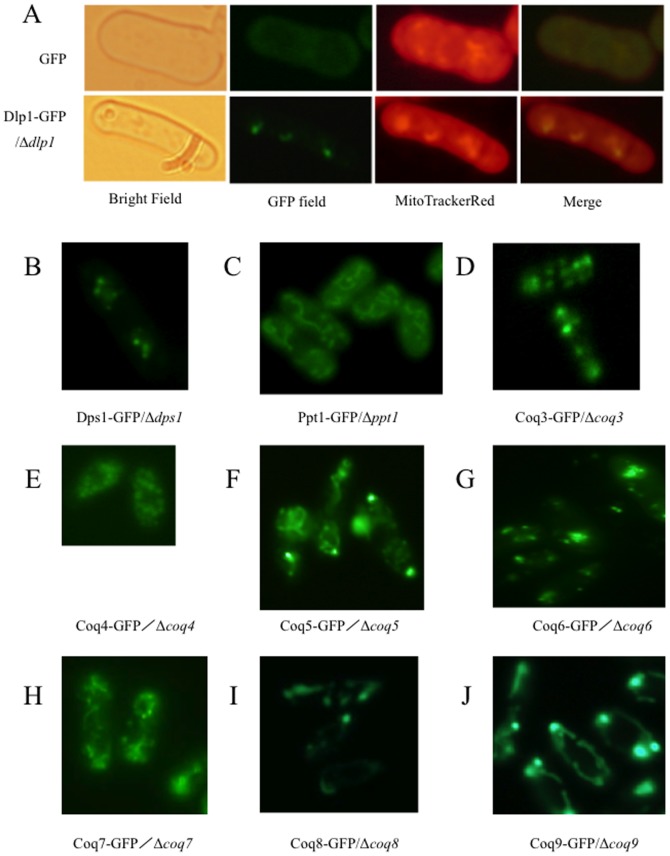
Mitochondrial localization of *S. pombe* Coq proteins. The indicated Coq proteins were fused with GFP and their localization was observed under a fluorescence microscope. The plasmids used in this experiment are shown in Figure S2B. (A) A bright-field image and the GFP and MitoTracker Red signals in the *Δdlp1* strain expressing the Dlp1-GFP fusion protein. The merged GFP and MitoTracker Red signals are also shown. (B–J) GFP signals of the Dps1-GFP fusion expressed in *Δdps1* cells (B), the Ppt1(Coq2)-GFP fusion expressed in *Δppt1* cells (C), the Coq3-GFP fusion expressed in *Δcoq3* cells (D), the Coq4-GFP fusion expressed in *Δcoq4* cells (E), the Coq5-GFP fusion expressed in *Δcoq5* cells (F), the Coq6-GFP fusion expressed in *Δcoq6* cells (G), the Coq7-GFP fusion expressed in *Δcoq7* cells (H), the Coq8-GFP fusion expressed in *Δcoq8* cells (I), and the Coq9-GFP fusion expressed in Δ*coq9* cells (J). All cells were stained with MitoTracker Red to verify the localization of the Coq proteins to mitochondria (data not shown).

The cellular localizations of Ppt1/Coq2 and Coq7 from *S. pombe* have been reported previously [20]. GFP-fusions were constructed to examine the localization of these and other *S. pombe* Coq proteins (Figure S3). As shown in Figure 3A, the fluorescent signal generated by the Dlp1-GFP fusion protein expressed in the *Δdlp1* strain overlapped with that of mitochondria stained using MitoTracker Red FM dye. The Dps1, Ppt1/Coq2, Coq3, Coq4, Coq5, Coq6, Coq7, Coq8, and Coq9 GFP fusion proteins also localized to the mitochondria (Figures 3B–J).

### Complementation of the *S. pombe coq* deletion strains by human *COQ* genes

In an HPLC analysis, a lipid extract from the wild type *S. pombe* strain (PR110) yielded a major peak at 10 min, which was consistent with that of authentic CoQ10. To compare the biosynthetic pathways of CoQ in *S. pombe* and higher organisms, we determined whether defective CoQ10 production by *S. pombe coq* deletion strains could be functionally complemented by human genes encoding CoQ biosynthetic enzymes. Plasmids containing Hs*COQ* genes (which were identified on the basis of their sequence similarity to known *S. pombe coq* genes) (Figure 1 and Table 2) under the control of the *nmt1* promoter were generated (Figure S4). For some human *COQ* genes, the use of a weaker promoter (nmt1*) was required to avoid growth inhibition caused by high levels of expression. The plasmids were expressed in the corresponding *S. pombe* deletion strains and lipid extracts were analyzed by HPLC.

**Figure 4:**
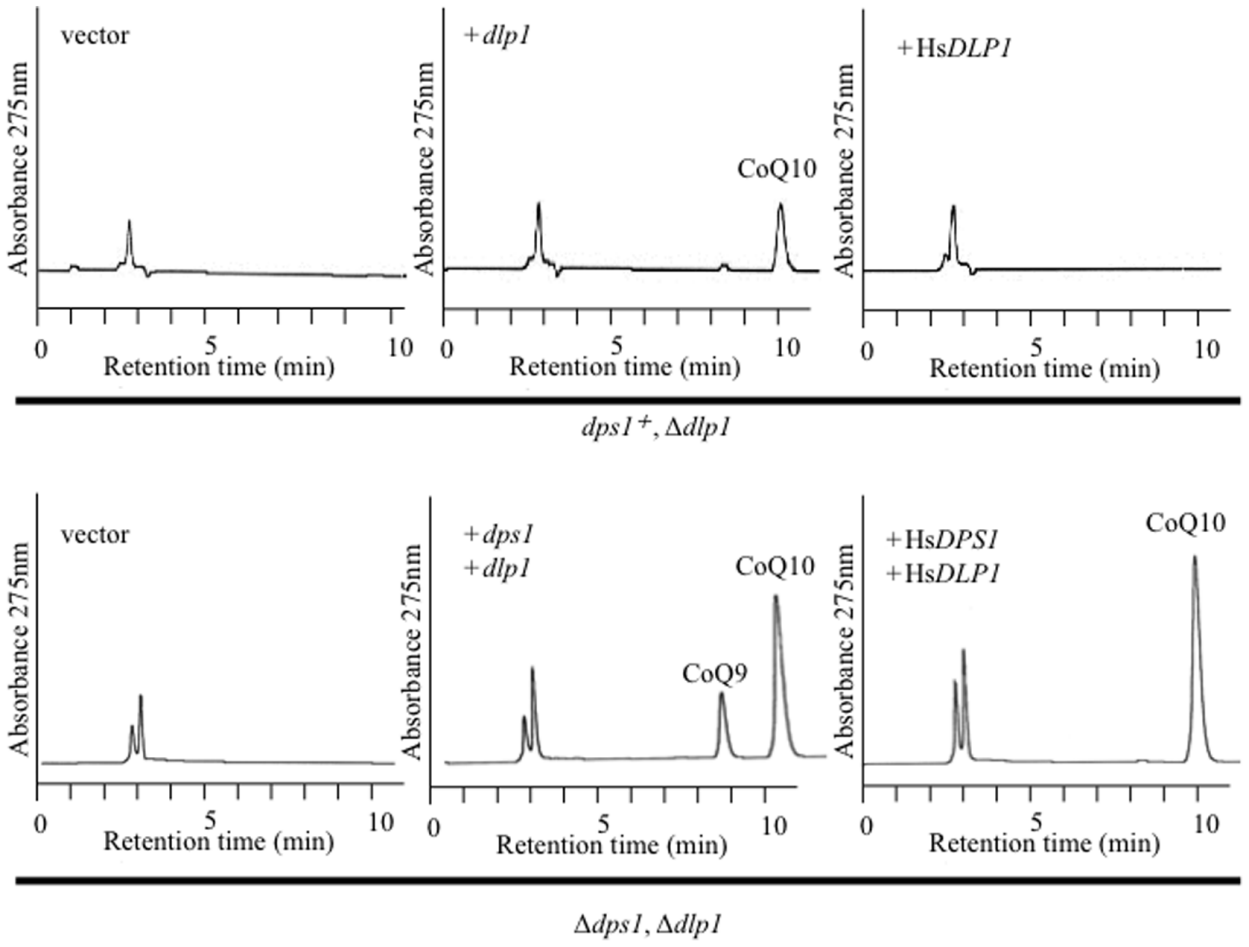
Functional complementation of the *S. pombe Δdps1* and *Δdlp1* strains by Hs*DPS1* and Hs*DLP1*. HPLC analyses of lipid extracts from the RM19 (*Δdlp1*) strain expressing *S. pombe dlp1* or Hs*DLP1* and the LA1 (*Δdps1-Δdlp1*) strain expressing *S. pombe dps1* and *dlp1* or Hs*DPS1* and Hs*DLP1*.

In a previous study, Hs*DPS1*/*PDSS1* was able to complement an *S. pombe Δdps1* mutant, but Hs*DLP1*/*PDSS2* was not able to complement an *S. pombe Δdlp1* mutant [12]. Here, the *S. pombe Δdlp1* strain expressing Hs*DLP1* containing a mitochondrial targeting signal from *dlp1* did not produce CoQ10 (Figure 4), indicating that the failure of complementation by this human gene is not due to mislocalization of the protein. By contrast, the *S. pombe dps1-dlp1* double deletion strain expressing Hs*DPS1* and Hs*DLP1* was able to produce CoQ10 (Figure 4), indicating that HsDPS1 and HsDLP1 can form a functional complex in *S. pombe*. These results suggest that, in both human and *S. pombe*, Dps1 and Dlp1 are necessary to constitute a heterotetrameric decaprenyl diphosphate synthase.

**Figure 5:**
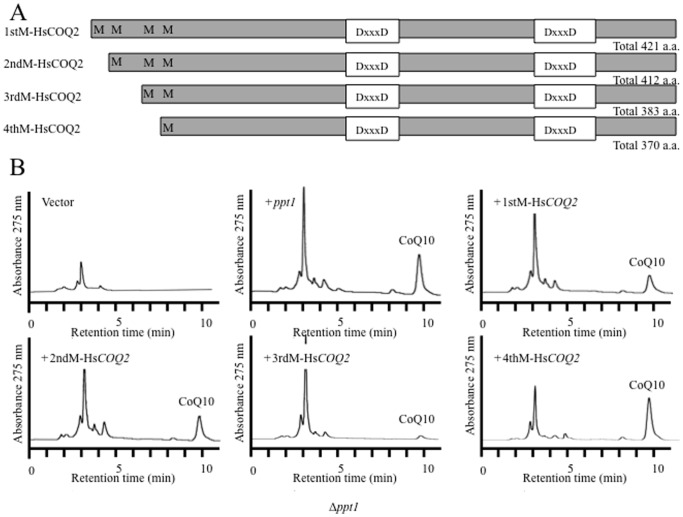
Functional complementation of the *S. pombe Δppt1* strain by Hs*COQ2*. (A) Schematic illustration of the four Hs*COQ2* plasmids that included different 5′ ends of the gene in pREP1. M, start codon; aa, amino acid. (B) HPLC analyses of lipid extracts from the KH2 (*Δppt1*) strain expressing *S. pombe ppt1* or the four different Hs*COQ2* constructs.

**Figure 6:**
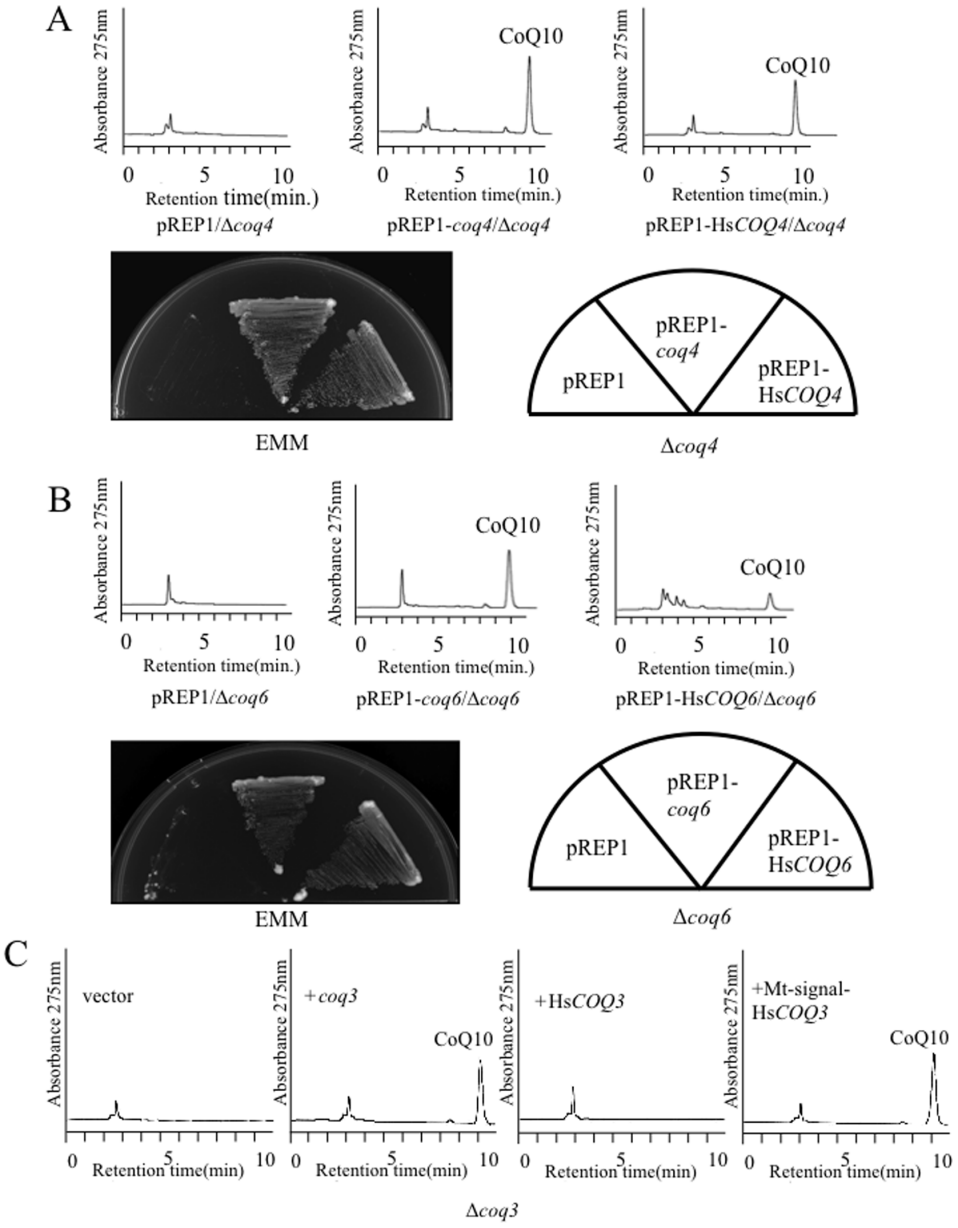
Complementation of the *S. pombe Δcoq4*, *Δcoq6* and *Δcoq3* strains by Hs*COQ3*, Hs*COQ4* and Hs*COQ6*. (A, B) HPLC analyses of lipid extracts from the KH4 (*Δcoq4*) strain expressing *S. pombe coq4* or Hs*COQ4* and the KH6 (*Δcoq6*) strain expressing *S. pombe coq6* or Hs*COQ6*. Growth on minimal media (EMM) plates is also shown. (C) HPLC analyses of lipid extracts from the KH3 (*Δcoq3*) strain expressing *S. pombe coq3*, Hs*COQ3*, or Hs*COQ3* fused with a mitochondrial targeting sequence (Mt-signal).

**Figure 7:**
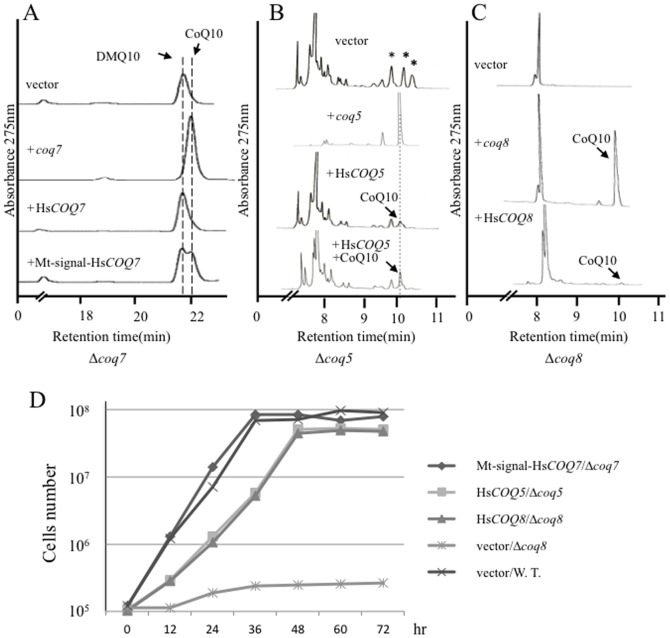
Complementation of the *S. pombe Δcoq7*, *Δcoq5,* and *Δcoq8* strains by Hs*COQ7,* Hs*COQ5* and Hs*COQ8*. (A) HPLC analyses of lipid extracts from the KH7 (*Δcoq7*) strain expressing *S. pombe coq7*, Hs*COQ7*, or Hs*COQ7* containing a mitochondrial targeting sequence (Mt-signal). The flow speed was doubled to separate the two peaks clearly. DMQ10, demethoxyubiquinone. (B) HPLC analyses of lipid extracts from the KH5 (*Δcoq5*) strain expressing *S. pombe coq5* or Hs*COQ5*. CoQ10 was mixed with the lipid extracts from the KH5 (*Δcoq5*) strain expressing Hs*COQ5*. Asterisks indicate the intermediate like peaks found in a *coq5* deletion strain. (C) HPLC analyses of lipid extracts from the KH8 (*Δcoq8*) strain expressing *S. pombe coq8* or Hs*COQ8*. (D) Cell growth of the indicated strains over 72 h. W.T., wild type strain.

Four putative start codons were identified in the Hs*COQ2* sequence; therefore, four plasmids that included a different putative start codon (1^st^–4^th^) of the gene were generated (Figure 5A). All of the Hs*COQ2* constructs restored the growth of the *S. pombe Δppt1* strain (data not shown) and recovered its defective production of CoQ10 (Figure 5B). Thus, the 4^th^ methionine is sufficient for functional expression in the *Δppt1* strain. Similar results were obtained when Hs*COQ4* and Hs*COQ6* were expressed in the *Δcoq4* and *Δcoq6* strains, respectively (Figures 6A and 6B). By contrast, Hs*COQ3* (Figure 6C) and Hs*COQ7* (Figure 7A) failed to complement their respective *S. pombe* deletion strains. Since all *S. pombe* Coq proteins localize to the mitochondria (Figure 3), the Hs*COQ3* and Hs*COQ7* constructs were modified to include a promoter region containing the mitochondrial targeting signal from the *S. pombe coq3* and *coq7* genes, respectively. Targeting of HsCOQ3 and HsCOQ7 to the mitochondria successfully recovered defective CoQ10 production by the *S. pombe Δcoq3* (Figure 6C) and *Δcoq7* (Figure 7A) strains. An HPLC peak that we previously identified as demethoxyubiquinone (DMQ10) [22] was detected in the *Δcoq7* strain; this peak was fully converted to CoQ10 by expression of *S. pombe coq7* and partly converted by expression of Hs*COQ7* containing a mitochondrial targeting signal (Figure 7A).

Unidentified peaks, which may represent intermediates, were identified in the *S. pombe Δcoq5* strain (Figure 7B, asterisks); these peaks did not merge with the CoQ10 standard (data not shown). When the *S. pombe coq5* gene was expressed in the *Δcoq5* strain, the intermediate peaks disappeared and a CoQ10 peak was observed on the HPLC trace (Figure 7B). By contrast, expression of the Hs*COQ5* gene in this strain failed to recover a clear CoQ10 production (Figure 7B); however, when the lipid extract from the Hs*COQ5*-complemented *Δcoq5* strain was mixed with CoQ10, one of the peaks merged with that of CoQ10, indicating that a small amount of CoQ10 was in fact produced (Figure 7B). Similarly, CoQ10 production by the *S. pombe Δcoq8* strain was fully recovered by expression of *S. pombe coq8* but only moderately recovered by expression of Hs*COQ8/ADCK3* (Figure 7C). Expression of Hs*COQ7* containing a mitochondrial targeting signal from *coq7* fully restored the growth of the *Δcoq7* strain, and Hs*COQ5* and Hs*COQ8/ADCK3* partially restored growth of the *Δcoq5* and *Δcoq8* strains, respectively; the doubling times of these complemented strains were approximately 0.7 times that of the wild type strain (Figure 7D). Despite the inclusion of a mitochondrial targeting signal within its sequence, Hs*COQ9* did not complement the *S. pombe Δcoq9* mutant (data not shown). Overall, these data indicate that, like the *S. pombe Δdps1*, *Δdlp1*, *Δppt1*/*coq2*, *Δcoq3*, *Δcoq7*, and *Δcoq8* strains described previously, the *Δ*c*oq4*, *Δ*c*oq5*, *Δ*c*oq6*, and *Δ*c*oq9* strains are also unable to synthesize CoQ. Furthermore, with the exception of *COQ9*, human *COQ* biosynthetic genes can functionally complement their corresponding *S. pombe coq* mutants.

### Sulfide and CoQ production

**Figure 8:**
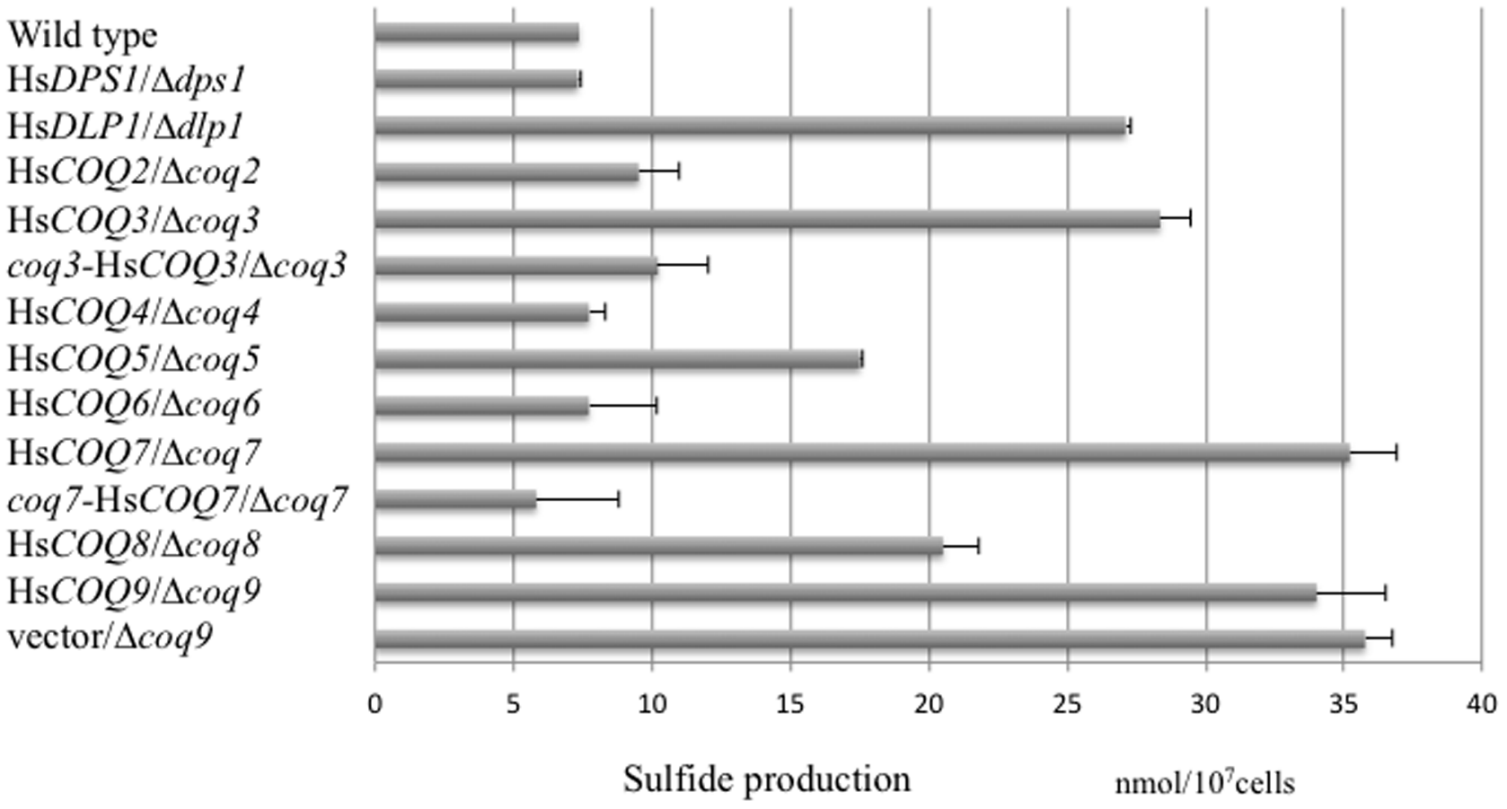
Sulfide production by *S. pombe coq* deletion strains expressing human *COQ* genes. Sulfide production was measured as described in the Materials and methods. Data are represented as the mean ± SD of n = 2 experiments. The *coq3*-Hs*COQ3*/*Δcoq3* and *coq7*-Hs*COQ7*/*Δcoq7* strains expressed the human genes containing a mitochondrial targeting sequence from *S. pombe coq3* and *coq7*, respectively.

We next measured sulfide production to further determine the efficiency of complementation of the *S. pombe coq* deletion strains by human *COQ* genes. Sulfide production in the *S. pombe coq* deletion strains is known to be higher than that in the wild type strain [6]. Expression of Hs*DPS1*, Hs*COQ2*, Hs*COQ4*, and Hs*COQ6* in the corresponding deletion strains reversed this increase (Figure 8). Expression of Hs*COQ5* and Hs*COQ8* (*ADCK3*) also partially reversed the increased sulfide production (Figure 8). Expression of Hs*COQ9* failed to reverse increased sulfide production by the *Δcoq9* strain. Similarly, expression of Hs*COQ3* and Hs*COQ7* failed to reverse increased sulfide production by the *Δcoq3* and *Δcoq7* strains, respectively; however, the addition of a mitochondrial targeting signal to these human genes successfully recovered sulfide production to a level similar to that of the wild type strain (Figure 8). These results are consistent with the levels of CoQ10 production in the human gene-complemented *coq* deletion strains, indicating that the restoration of CoQ10 production and sulfide production was correlated.

### Complementation of *S. pombe coq* deletion strains by *A. thaliana* genes

**Figure 9:**
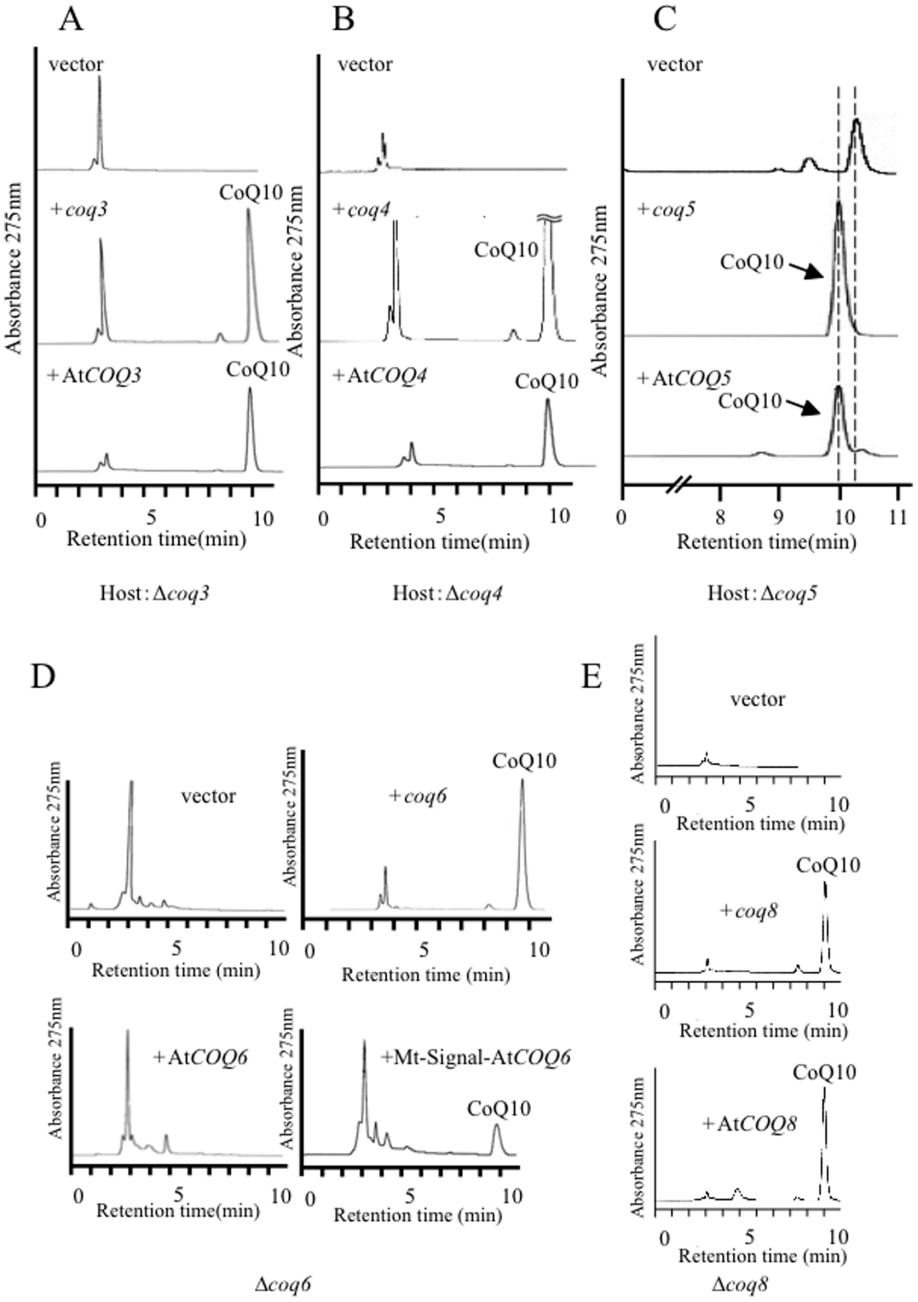
Complementation of the *S. pombe coq* deletion strains by *A. thaliana COQ* genes. (A–E) HPLC analyses of lipid extracts from the KH3 (*Δcoq3*) strain expressing *S. pombe coq3* or At*COQ3* (A); the KH4 (*Δcoq4*) strain expressing *S. pombe coq4* or At*COQ4* (B); the KH5 (*Δcoq5*) strain expressing *S. pombe coq5* or At*COQ5* (C); the KH6 (*Δcoq6*) strain expressing *S. pombe coq6*, At*COQ6*, or At*COQ6* fused with a mitochondrial targeting sequence (Mt-Signal) (D); and the KH8 (*Δcoq8*) strain expressing *S. pombe coq8* or At*COQ8* (E). DMQ10, demethoxyubiquinone.

Next, we examined the ability of *A. thaliana* (At) *COQ* genes (Table 2) to complement the *S. pombe coq* deletion mutants. Although At*SPS1* and At*SPS2*, which encode two solanesyl diphosphate synthases in *A. thaliana*, complement the fission yeast *Δdps1* mutant [13], recent studies show that At2g34630 (named here AtSPS3) is most likely responsible for CoQ synthesis in *A. thaliana* [39]. Here, the abilities of the At*COQ3*, At*COQ4*, At*COQ5*, At*COQ6*, and At*COQ8* genes to complement the corresponding *S. pombe* mutants were determined. Expression of At*COQ3*, At*COQ4* and At*COQ5* recovered CoQ10 production by the *S. pombe Δcoq3*, *Δcoq4* and *Δcoq5* strains, respectively (Figures 9A–C). By contrast, expression of the At*COQ6* gene did not complement the *Δcoq6* strain; however, the addition of a mitochondrial targeting signal to At*COQ6* did enable recovery of CoQ10 production when expressed in the *Δcoq6* strain (Figure 9D). *A. thaliana* does not contain an ortholog of Coq7, and both Coq6 and Coq7 are involved in the hydroxylation step of CoQ biosynthesis; however, At*COQ6* was unable to recover CoQ10 production by the *S. pombe Δcoq7* strain (data not shown). It is still not clear what kind of enzyme is responsible for this hydroxylation step in *A. thaliana*. Unlike Hs*COQ8*/*ADCK3*, the At*COQ8* gene was able to functionally complement the *S. pombe Δcoq8* strain (Figure 9E); however, expression of At*COQ9* did not recover CoQ10 production by the *Δcoq9* strain (data not shown).

## Discussion

Ten *coq* deletion strains of the fission yeast *S. pombe*, including six that were reported previously (Uchida, *et al*., 2000, Saiki, *et al*., 2003, Miki, *et al*., 2008), were created. The ten deletion strains were phenotypically indistinguishable; they did not produce CoQ10 (were respiration defective), were sensitive to oxidative stress, produced a large amount of sulfide, required an antioxidant to grow on minimal medium, were sensitive to Cu^2+^, and did not survive long at the stationary phase. In recent large scale deletion library screening studies, a number of *coq* deletion strains were sensitive to Cd^2+^ and doxorubicin, an inhibitor of topoisomerase 2 [40], [41]. As a consequence of the various roles of CoQ, *coq* deletion strains show pleiotropic phenotypes. CoQ is an essential component of the electron transfer system; therefore, *coq* deletion strains grew slowly under non-fermentation conditions and failed to grow on medium containing glycerol and ethanol as carbon sources. Since CoQ functions as an antioxidant and couples sulfide oxidation and cysteine metabolism, the *S. pombe coq* deletion strains were sensitive to H_2_O_2_ and produced large amounts of sulfide. CoQ is also required for *de novo* UMP synthesis, resulting in poor growth of the *coq* deletion strains on minimal medium [7]. We did not see the growth recovery of CoQ deficient *S. pombe* cells by supplementation of CoQ10 (Figure S6), whereas supplementation of CoQ6 generally restored the growth of *S. cerevisiae coq* deletion mutants on non-fermentable carbon source [42]. The slow growth of a *coq* deficient mutant was partly recused by supplementation of CoQ10 encapsulated by γ–cyclodextrin, but its respiration was not restored [43]. It is not easy to test the effect of CoQ10 supplementation in *S. pombe*. In *S. cerevisiae*, the two essential genes, *ARH1* and *YAH1*, are involved in CoQ synthesis through the regulation of Coq6 [26]. Although *S. pombe* contains orthologs of these genes, it will be difficult to determine their functions in CoQ biosynthesis via deletion analyses because they are essential for growth.

All *S. pombe coq* deletion strains failed to synthesize CoQ10, intermediate peaks were observed on HPLC traces of lipid extracts from the *Δcoq5* and *Δcoq7* strains. The peak identified in the *Δcoq7* extract was previously determined to be DMQ10 [22], while those identified in the *Δcoq5* extract are currently unknown. At least two peaks were detected in the *Δcoq5* extract, which may indicate the existence of two pathways originating from the precursors PHB and pABA, as reported in *S. cerevisiae* [18]; however, additional studies are required to confirm this hypothesis.

The results presented here demonstrate that all of the *S. pombe* Coq proteins localize to mitochondria, indicating that CoQ biosynthesis occurs in these cellular compartments. These results are consistent with those of a previous study [44] that examined the localization of 4,431 *S. pombe* proteins, although Coq8 was not included. Coq proteins in *S. cerevisiae* also localize to mitochondria, where they form a complex [23]. A recent study showed that biosynthesis of CoQ in humans also occurs in the Golgi [45], where one of the CoQ biosynthetic steps, PHB-polyprenyl condensation, is mediated by the enzyme UBIAD1. Because this enzyme is absent in yeasts, it is likely that CoQ is synthesized predominantly in mitochondria in *S. cerevisiae* and *S. pombe*.

As mentioned above, Hs*DPS1*/*PDSS1* is able to complement an *S. pombe Δdps1* mutant, but Hs*DLP1*/*PDSS2* is not able to complement an *S. pombe Δdlp1* mutant [12]. This suggests that the *S. pombe* Dps1 protein cannot form a complex with the HsDLP1 protein, even though they share sequence similarity (28% identity; see Table 3). When Hs*DPS1* and Hs*DLP1* genes were expressed in a *Δdps1 Δdlp1* strain, CoQ10 was predominantly produced, while *S pombe* naturally produce CoQ10 with a lesser amount of CoQ9 (Fig. 4). This result indicates decaprenyl diphosphate synthase from humans strictly produces 10 isoprene units of prenyl diphosphate, which is consistent with the observation that CoQ10 is predominantly found in human bodies.

**Table 3:** Amino acid sequence similarity of Coq proteins from *S. pombe*, *S. cerevisiae,* humans, and *A. thaliana.*

*Schizosaccharomyces pombe*	SpDps1 (378 aa)	SpDlp1 (294 aa)	SpPpt1 (360 aa)	SpCoq3 (274 aa)	SpCoq4 (272 aa)	SpCoq5 (305 aa)	SpCoq6 (479 aa)	SpCoq7 (216 aa)	SpCoq8 (610 aa)	SpCoq9 (250 aa)	SpCoq10 (164 aa)
*Saccharomyces cerevisiae*	ScCoq1 (473 aa)	—	ScCoq2 (372 aa)	ScCoq3 (312 aa)	ScCoq4 (335 aa)	ScCoq5 (307 aa)	ScCoq6 (479 aa)	ScCoq7 (233 aa)	ScCoq8 (501 aa)	ScCoq9 (260 aa)	ScCoq10 (207 aa)
Identity	37%	—	48%	35%	43%	53%	36%	51%	50%	29%	30%
Positive	53%	—	65%	51%	62%	69%	58%	66%	69%	53%	49%
*Homo sapiens*	HsPDSS1 (415 aa)	HsPDSS2 (399 aa)	HsCOQ2 (371aa)	HsCOQ3 (369 aa)	HsCOQ4 (265 aa)	HsCOQ5 (327 aa)	HsCOQ6 (468 aa)	HsCOQ7 (217 aa)	HsCOQ8/ADCK3 (595 aa)	HsCOQ9 (318 aa)	HsCOQ10A (230 aa)
Identity	45%	28%	48%	38%	44%	47%	34%	45%	46%	32%	31%
Positive	62%	49%	63%	54%	64%	63%	49%	64%	62%	53%	47%
*Arabidopsis thaliana*	AtSPS3 (423 aa)	—	AtPPT1 (407 aa)	AtCOQ3 (322 aa)	AtCOQ4 (226 aa)	AtCOQ5 (288 aa)	AtCOQ6 (505 aa)	—	AtCOQ8 (623 aa)	AtCOQ9 (311 aa)	AtCOQ10 (256 aa)
Identity	38%	—	47%	40%	39%	49%	29%	—	39%	28%	28%
Positive	60%	—	61%	58%	58%	62%	46%	—	57%	48%	49%

**Figure 10:**
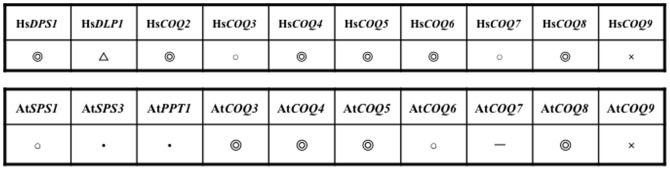
Summary of complementation of *S. pombe coq* deletion strains by human and *A. thaliana* genes. The double open circles indicate genes that complemented the *S. pombe* deletion mutants. The single open circles indicate genes that complemented the *S. pombe* deletion mutants after fusion of a mitochondrial targeting sequence. Hs*DLP1* is only functional in the presence of Hs*DPS1*, so is denoted by a triangle. The “x” symbols indicate genes that failed to complement the *S. pombe* deletion mutants. *A. thaliana* does not contain a *COQ7* ortholog. Closed circles indicate genes that complemented the corresponding *S. cerevisiae* mutants but were not tested in *S. pombe*.

Homologues of all of the *S. pombe coq* genes exist in humans and homologues of all except *coq7* exist in *A. thaliana*. In most cases, functional complementation of the *S. pombe coq* deletion strains by human or *A. thaliana* genes was successful (Figure 10); however, in some cases (Hs*COQ3*, Hs*COQ7* and At*COQ6*), the addition of an N-terminal mitochondrial targeting sequence from the corresponding *S. pombe coq* gene was required for complementation. One of reasons may be a shortage of targeting sequences in cases of Hs*COQ3* and Hs*COQ7*, as there are longer length transcriptional variants in these genes. The other reason may be incapability of working mitochondrial targeting sequence of humans or *A. thaliana* genes in *S. pombe* cells. The sequences of the N-terminal regions of the *coq*/*COQ* genes in *S. pombe*, humans, and *A. thaliana* are rather diverged, and we were unable to find any apparent rules that govern how the N-terminal sequences affect mitochondrial targeting.

Although some complementation analyses of *S. cerevisiae coq* deletion strains expressing human genes have been conducted, a comprehensive analysis has not yet been performed. Studies performed to date show that expression of Hs*COQ2* [46], Hs*COQ3* [21], Hs*COQ4* [47], and Hs*COQ6* [48] functionally complement their corresponding *S. cerevisiae coq* mutants. Hs*COQ8* (*ADCK3*) also complements the *S. cerevisiae Δcoq8* mutant weakly [49], which is consistent with the results presented here for *S. pombe*. To our knowledge, functional complementation of *S. cerevisiae Δcoq5* and *Δcoq7* mutants by human *COQ5* or *COQ7*, respectively, has not yet been tested. AtSPS3, which encodes solanesyl diphosphate synthase, and At*PPT1*, which encodes PHB-polyprenyl diphosphate transferase, complement the *S. cerevisiae Δcoq1* and *Δcoq2* mutants, respectively [9], [13].

Hs*COQ9* and At*COQ9* failed to complement the *S. pombe Δcoq9* strain. Similarly, in a previous study, Hs*COQ9* failed to complement the *S. cerevisiae Δcoq9* mutant [50]. Although its exact function is still unknown, Coq9 is absolutely required for the biosynthesis of CoQ in *S. cerevisiae* and, as shown here, *S. pombe*. Genetic disorders of CoQ biosynthesis related to mutation of the Hs*COQ9* gene [51] suggest that this enzyme is also involved in CoQ synthesis in humans. However, the function of COQ9 may not be conserved between humans, *A. thaliana,* and yeast because these Coq9 proteins share only 28–30% amino acid identity (Table 3).

Complementation analyses of plant genes encoding CoQ biosynthetic enzymes are scare. Based on an analysis of the At*PPT1* gene, CoQ appears to be essential for seed formation in *A. thaliana* [9]. At*SPS1* is functional when expressed in *S. pombe* [13] but the protein may not be involved in CoQ synthesis; on the other hand, AtSPS3 (*At2g34630*) is responsible for CoQ synthesis in this species [39]. Since *A. thaliana* does not contain a *COQ7* gene, we tested the ability of the other *COQ* genes in this species (At*COQ3*, At*COQ4*, At*COQ5*, At*COQ6*, At*COQ8*, and At*COQ9*) to recover CoQ10 production in *S. pombe coq* deletion strains. With the exception of AtCOQ9, the functions of all other *A. thaliana* COQ proteins were conserved. In knockdown analyses of *Caenorhabditis elegans*, the *coq7* deletion strain was able to survive for long periods, unlike other *coq* deletion strains. No apparent Coq9 ortholog was found in *C. elegans*, which might be relevant to the fact this organism produces rhodoquinone.

In conclusion, this study describes the functional conservation of CoQ biosynthetic genes in humans, plants, and the fission yeast *S. pombe*. It also demonstrates that the functions of the human *COQ* genes can be determined by expression in *S. pombe* deletion mutants. Determining the function of CoQ biosynthetic genes may be useful for understanding genetic disorders caused by human CoQ biosynthetic deficiencies.

## Supporting Information

Figure S1 **Construction of the** ***S. pombe ppt1, coq4, coq5, coq6, coq8*** **, and** ***coq9*** **deletion strains.** One step homologous recombination was used to delete the *S. pombe coq* genes. The *coq4* deletion strategy is shown as an example. The coq4-x and coq4-y primers were homologous to the flanking regions of the *coq4* and *kan* resistance genes. The coq4-w and coq4-z primers were homologous to regions located approximately 500 bp downstream and upstream of the *coq4* gene. The nb2 and coq4-c primers were used to verify the replacement of *coq4* by *kan*. All other deletion strains were constructed similarly.
(TIFF)

Figure S2 **Construction of the plasmids to express** ***S. pombe coq*** **genes.** The *S. pombe coq* genes were inserted into the pREP1 or pREP2 vector under the control of the *nmt1* promoter.
(TIFF)

Figure S3 **Construction of the plasmids to express** ***S. pombe coq-GFP*** **.** To determine the cellular localization of Coq proteins, GFP-fusions were generated by inserting the *coq* genes into the pSFL272L-GFP_S65A_ vector (used in most cases).
(TIFF)

Figure S4 **Construction of the plasmids to express human** ***COQ*** **genes.** The human *COQ* genes were inserted into the pREP1, pREP41, or pREP2 vectors. The promoter region and mitochondrial targeting signal of *dlp1, coq3* or *coq7* from *S. pombe* were fused with the Hs*DLP1*, Hs*COQ3* or Hs*COQ7* gene, respectively.
(TIFF)

Figure S5 **Construction of the plasmids to express** ***Arabidopsis COQ*** **genes.** The plasmids containing the *A. thaliana COQ* genes were constructed in the same manner as those expressing the human *COQ3* gene. The *S. pombe coq6* or *coq9* mitochondrial targeting sequence was fused with the At*COQ6* or At*COQ9* gene, respectively.
(TIFF)

Figure S6 **Growth profile of a** ***dps1*** **deletion strain with supplementation of CoQ10.** Cell growth of the Δ*dps1* strain (LJ1030) in YES or EMM suppemented with 10 µM CoQ10 (final concentration) dissolved in ethanol was monitored by the cell counter (Sysmex Corp.) over 72 h.
(TIFF)

Table S1 **Primers used in this study.** Primers used for construction of various plasmids and deletion strains are listed.
(DOCX)
