# Supplementary figures and images for: Functional Conservation of Coenzyme Q Biosynthetic Genes among Yeasts, Plants, and Humans
Source: PLoS One. 2014 Jun 9;9(6):e99038. doi: 10.1371/journal.pone.0099038 (PMC4049637; doi:10.1371/journal.pone.0099038)

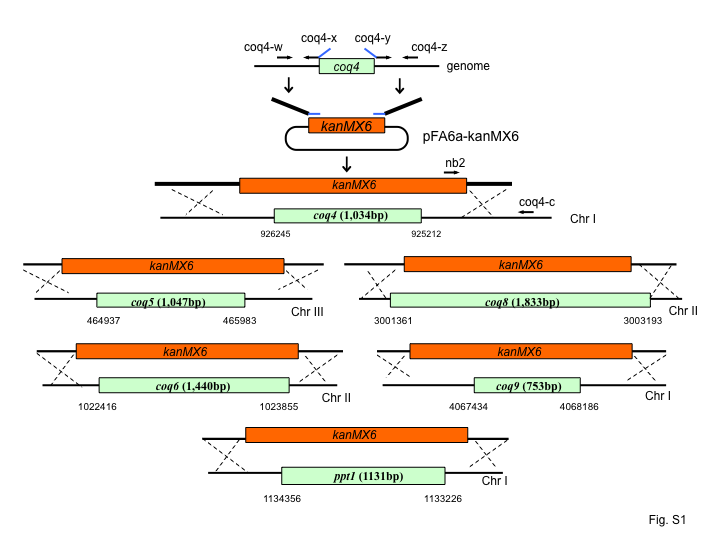

Supplement: Figure S1 — Construction of the S. pombe ppt1, coq4, coq5, coq6, coq8 , and coq9 deletion strains. One step homologous recombination was used to delete the S. pombe coq genes. The coq4 deletion strategy is shown as an example. The coq4-x and coq4-y primers were homologous to the flanking regions of the coq4 and kan resistance genes. The coq4-w and coq4-z primers were homologous to regions located approximately 500 bp downstream and upstream of the coq4 gene. The nb2 and coq4-c primers were used to verify the replacement of coq4 by kan. All other deletion strains were constructed similarly. (TIFF) [file pone.0099038.s001.tif]

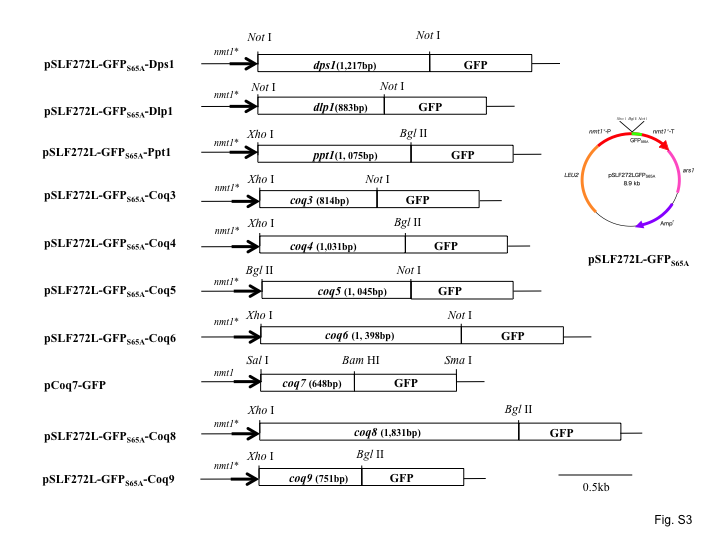

Supplement: Figure S3 — Construction of the plasmids to express S. pombe coq-GFP . To determine the cellular localization of Coq proteins, GFP-fusions were generated by inserting the coq genes into the pSFL272L-GFPS65A vector (used in most cases). (TIFF) [file pone.0099038.s003.tif]
