# Supplementary material for: Functional Conservation of Coenzyme Q Biosynthetic Genes among Yeasts, Plants, and Humans
Source: PLoS One. 2014 Jun 9;9(6):e99038. doi: 10.1371/journal.pone.0099038 (PMC4049637; doi:10.1371/journal.pone.0099038)
Supplement: Table S1 — Primers used in this study. Primers used for construction of various plasmids and deletion strains are listed. (DOCX) [file pone.0099038.s007.docx]

Table S1. Primers used in this study

| Name | Sequence |
| --- | --- |
| pFA6a-F | 5’-TCGTACGCTGCAGGTCGACG-3’ |
| pFA6a-R | 5’-CATCGATGAATTCGAGCTCG-3’ |
| Nb2 | 5’-GTTTAAACGAGCTCGAATTC-3’ |
| Dps1-N-GFP (NotI) | 5’-TCGCGGCCGCATGATTCAGTAT-3’ |
| Dps1-C-GFP (NotI) | 5’-CTGCGGCCGCACTTCTTTCTCGT-3’ |
| Dlp1-N (SalI) | 5’-tcgtcgacgagctttccgttc-3’ |
| Dlp1-C (SmaI) | 5’-tcccgggattacttcgaaAc-3’ |
| Dlp1-N-GFP (NotI) | 5’-TCGCGGCCGCATGAGCTTTCCG-3’ |
| Dlp1-C-GFP (NotI) | 5’-CTGCGGCCGCACTTCGAAACACTATTAGCG-3’ |
| Ppt1-N-GFP (Xho1) | 5'-CCGCTCGAGAATGATAATTAAGC-3' |
| Ppt1-C-GFP (BglII) | 5'-CGAGATCTCCAGAATCGTAAAT-3' |
| ppt1-w | 5'-GAATCGTCTGGCTCTCAAATAC-3' |
| ppt1-x | 5'-GGGGATCCGTCGACCTGCAGCGTACGATCTCTATACTACAATTAC-3' |
| ppt1-y | 5'-GTTTAAACGAGCTCGAATTCATCGATCATATCATACCAGATGTG-3' |
| ppt1-z | 5'-CCTGAAAATCTTGAGCACTG-3' |
| ppt1-c | 5'-GGTCAAATCTCCGTTTTAGAGG-3' |
| Coq3-N (SalI) | 5'-acgcgtcgacgaactcaatgaacattc-3' |
| Coq3-C (SmaI) | 5'-tcccccgggcgaatcaggcagacatgg-3' |
| Coq3-N-GFP (Xho I) | 5’-ccgctcgagaatgaacattctc-3’ |
| Coq3-C-GFP (NotI) | 5’-ttgcggccgcgggcagacatgg-3’ |
| Coq4-N (Bam HI) | 5’-CCCGGATCCTATGTTTTATCTTAATGCTCATC-3’ |
| Coq4-C (Sma I) | 5’-GGCCCGGGCTAAACCAGCGG-3’ |
| Coq4-N-GFP (Xho I) | 5’-CCGCTCGAGAATGTTTTATCTTAAT-3’ |
| Coq4-C-GFP (Bgl II) | 5’-AATAGATCTGGAACCAGCGGAGGAG-3’ |
| coq4-w | 5’-CACTAATATCAGGAAACATGAG-3’ |
| coq4-x | 5’-GGGGATCCGTCGACCTGCAGCGTACGAGTGAAGTTCAGTATTGC-3’ |
| coq4-y | 5’-GTTTAAACGAGCTCGAATTCATCGATCCATTTACTCAATCG-3’ |
| coq4-z | 5’-GTTTTTCGTGGAATTTAGGC-3’ |
| coq4-c | 5’-TGCGTACTATTAAGCGTCTGC-3’ |
| Coq5-N (SalI) | 5’-CTGCGTCGACATGTCACGTTTAAG-3’ |
| Coq5-C (Bam HI) | 5’-GCTGGATCCCCATCCTACAAC-3’ |
| Coq5-N-GFP (Bgl II) | 5’-CCGAGATCTCATGTCACGTT-3’ |
| Coq5-C-GFP (NotI) | 5’-TTGCGGCCGCGCAACTTGATAC-3’ |
| coq5-w | 5’-TCAATTTTCTTATCATTTCCC-3’ |
| coq5-x | 5’-GGGGATCCGTCGACCTGCAGCGTACGAAATACCAAGTGTAACGG-3’ |
| coq5-y | 5’-GTTTAAACGAGCTCGAATTCATCGATACTGCAAAGGCATAATGC-3’ |
| coq5-z | 5’-ATTGTTTAATGTGGTTGCTGTGC-3’ |
| coq5-c | 5’-TAAAGGACCTGAAGAGCTC-3’ |
| Coq6-N (SalI) | 5’-ggggtcgactatgcctactatcagtcc-3’ |
| Coq6-C (SmaI) | 5’-ggcccgggctaaattccattagc-3’ |
| Coq6-N-GFP (XhoI) | 5’-AGCCTCGAGAATGCCTACTATC-3’ |
| Coq6-C-GFP (NotI) | 5’-TTGCGGCCGCCAATTCCATT-3’ |
| coq6-w | 5’-GCTTCACTTTTAGTAGCAAATGC-3’ |
| coq6-x | 5’-GGGGATCCGTCGACCTGCAGCGTACGACCAAAGAACTCATGAGCC-3’ |
| coq6-y | 5’-GTTTAAACGAGCTCGAATTCATCGATTACCACTGGAGCTGAC-3’ |
| coq6-z | 5’-GCCAGTACATTGCTTGATTC-3’ |
| coq6-c | 5’-TGCTAGTCCAGCTGAAGTC-3’ |
| coq8-N (XhoI/NdeI) | 5'- CTCGAGCATATGGCTAATTCAGGG-3' |
| coq8-C (SmaI) | 5'-CCCGGGTTTAATCAGCGTAATG-3' |
| Coq8-N-GFP (XhoI) | 5’-CCGCTCGAGAATGGCTAATTC-3’ |
| Coq8-C-GFP (BglII) | 5’-CCGAGATCTCCATCAGCGTAATG-3’ |
| coq8-w | 5’-gtacgatagtaaaggtaaaaatg-3’ |
| coq8-x | 5’-ggggatccgtcgacctgcagcgtacgaggaagggctttcaagtg-3’ |
| coq8-y | 5’-gtttaaacgagctcgaattcatcgatcacattggttgaaaccct-3’ |
| coq8-z | 5’-gaaagtctgcttcaaaggc-3’ |
| coq8-c | 5’-cgatagataaaggacatcac-3’ |
| Spcoq9-N (NdeI) | 5'-GGCCCCATATGTTGACTTTAAGA-3' |
| Spcoq9-C (BamHI) | 5'-CGGATCCTTAAATTCCCCTTGA-3' |
| Coq9-N-GFP (Xho1) | 5'-CCGCTCGAGCATGTTGACTTTAAGA-3' |
| Coq9-C-GFP (BglII) | 5'-CGAGATCTCCAATTCCCCTTGAACG-3' |
| coq9-w | 5'-GGCATTCGGGGAACATAC-3' |
| coq9-x | 5'-GGGGATCCGTCGACCTGCAGCGTACGAGCACTTCTTAAAGTCAAC-3' |
| coq9-y | 5'-GTTTAAACGAGCTCGAATTCATCGATAATCCAACGTTCAAGGGG-3' |
| coq9-z | 5'-GGCCTTGTAGAGGTATAG-3' |
| coq9-c | 5'-CCCGGATCCTTAAAGATCTCCAATTCCCCTTGAACG-3' |
| DlpTP-N (SalI) | 5'-CCGTCGACTACTTACTATTCTGACTC-3' |
| DlpTP-C (NdeI) | 5'-CCCCATATGGGAACCAGGTTTTTTTA-3' |
| HsDLP-N (NdeI) | 5'-CCCCATatgaactttcggcagctgct-3' |
| HsDLP-C (BamHI) | 5'-CGGATCCTCATGAAAATCTGG-3' |
| HsCOQ2-1stMet (SalI) | 5'-GGTCGACTATGACCCCAATTTCACAAGTAAGGATGAGG-3' |
| HsCOQ2-2ndMet (SalI) | 5'-GAAGTCGACAATGAGGAAAGGTTCTGCCCACACCGCC-3' |
| HsCOQ2-3rd Met (SalI) | 5'-aagtcgacaatgacgtcaatccg-3' |
| HsCOQ2-4thMet (SalI) | 5'-5'-CCGTCGACCATGCTGGGCTCGCGAGC-3' |
| HsCOQ2-C (SmaI) | 5'-cccccgggttaattttctatttt-3' |
| HsCOQ3-N (NdeI) | 5'-ccagaaccatatgttcaaatcc-3’ |
| HsCOQ3-C (BamHI) | 5'-gaggatcctcatttcttcaga-3’ |
| Sp3TP-N (BamHI) | 5'-GGGATCCCTTAACTTGTTTACGACAACT-3' |
| Sp3TP-C (BglII) | 5'-CCAGATCTATTTACTGACACTGAATG-3' |
| HsCOQ3-N (BglII) | 5'-CCAGATCTATGTTCAAATCCTACAGGAC-3' |
| HsCOQ3-C (Sma1) | 5'-CCCCCGGGTCATTTCTTCAGCTTTT-3' |
| HsCOQ4-N (NdeI) | 5’-ATCCGCTCATATGGCGACTCTG-3’ |
| HsCOQ4-C (SalI) | 5’-ATTGGCTCGTCGACTCAGGCCAAG-3’ |
| HsCOQ5-N (NdeI)) | 5’-TCGCGGCATATGCGGGGCTGCCAG -3’ |
| HsCOQ5-C (NdeI)) | 5’- ATAGGACATATGTTAAAGTTTGAAGCC-3’ |
| HsCOQ6-N (NdeI) | 5’-AATTCTGCCATATGGCGGCCC-3’ |
| HsCOQ6-C (SalI) | 5’-GAGAGGTCGACTCATTTGCTTGC-3’ |
| HsCOQ7-N (SalI) | 5’-acgcgtcgacaatgactttagac-3’ |
| HsCOQ7-C (BamHI) | 5’-cgcggatccgacacacttataatc-3’ |
| Coq7-TP35-N (XhoI) | 5’-ccgctcgagagtctctacttgtacttg-3’ |
| Coq7-TP35-C (NotI) | 5’-ttgcggccgcccagttctttacggcc-3’ |
| HsCOQ7-N (HindIII) | 5’-cccaagcttatgactttagac-3’ |
| HsCOQ8-N (NdeI) | 5’-ccctgacatatggctgccatattg-3’ |
| HsCOQ8-C (NdeI) | 5’-cccatatgctactgctgggcctg-3’ |
| HsCOQ9-N (SalI) | 5'-GTCGACTATGGCGGCGGCGGCGGTATC-3' |
| HsCOQ9-C (BamHI) | 5'-GGATCCTCACCGACGCTGGTTTAG-3' |
| Sp9TP-N (SalI) | 5'-GGGTCGACTATGTTGACTTTAAGAAG-3' |
| Sp9TP-HsCOQ9-C | 5'-GATACCGCCGCCGCCGCCATAATTAAAGCCTTCTTTCCGG-3' |
| HsCOQ9-N | 5'-ATGGCGGCGGCGGCGGTATC-3' |
| AtCOQ3-N (SalI) | 5'-GTCGACTATGTTGGCGTC-3' |
| AtCOQ3-C (SmaI) | 5'-CCCGGGTTATATGTCTCC-3' |
| AtCOQ4-N (Sal1) | 5'-GTCGACTATGATTATTGAAAG-3' |
| AtCOQ4-C (BamHI) | 5'-CGGATCCTCAAATGCGAGTAC-3' |
| AtCOQ5-N (NdeI) | 5'-ccccAtatggcactacga-3' |
| AtCOQ5-C (BamHI) | 5'-cggatcctcagagctttatg-3’ |
| AtCOQ6-N (NdeI) | 5'-CCCCATATGAACAGGGTGCTC-3' |
| AtCOQ6-C (BamHI) | 5'-CCGGATCCTCAAGAAAATAGCG-3' |
| Sp6TPAtCOQ6-N (NdeI) | 5’-CCCATATGCCTACTATCAGTCCAACTTTAGGAATATACACGAGGAAGTTTGCAAGCCAAAAAATACTTCAACGTCAATTCATGAACAGGGTGCTCGC-3’ |
| AtCOQ8-N (NdeI) | 5'-CCCATATGAGTTCGTGGAGC-3' |
| AtCOQ8-C (SalI) | 5'-GGGTCGACCTATGAAGAAACAGAGC-3' |
| AtCOQ9-N (NdeI) | 5'-CCCATATGTACCGAACGGCG-3' |
| AtCOQ9-C (SalI) | 5'-GGTCGACTCAAAACGCAGAGC-3' |
| Sp9TP-AtCOQ9-C | 5'-CGCCGCCGTTCGGTACATAATTAAAGCCTTCTTTCCGG-3' |
